# Comparative effects of alfentanil-remimazolam versus fentanyl-remimazolam on anesthesia onset, emergence, and safety in first-trimester surgical abortion under intravenous anesthesia: a randomized controlled trial

**DOI:** 10.3389/fmed.2026.1796064

**Published:** 2026-04-08

**Authors:** Weijie Zhang, Jiao Wang, Kangning Yang, Xiaoyi Pang

**Affiliations:** Department of Anesthesiology, Mianyang 404 Hospital, Mianyang, Sichuan, China

**Keywords:** alfentanil-remimazolam, anesthesia induction, emergence time, fentanyl-remimazolam, somatomotor response, surgical abortion

## Abstract

**Background:**

Alfentanil is a short-acting *μ*-opioid receptor agonist that can be used synergistically with other sedatives. This study aimed to investigate the clinical efficacy of an alfentanil-remimazolam combination for first-trimester surgical abortion (vacuum aspiration) under intravenous anesthesia.

**Methods:**

A total of 120 patients undergoing first-trimester surgical abortion under intravenous anesthesia at our hospital were recruited between January 1, 2025 and June 30, 2025, and were randomly assigned to two groups: the alfentanil-remimazolam group (AF-RMZ, *n* = 60) and the fentanyl-remimazolam group (F-RMZ, *n* = 60). The AF-RMZ group received alfentanil (10 μg kg^−1^) combined with remimazolam (0.3 mg kg^−1^), while the F-RMZ group received fentanyl (1 μg kg^−1^) combined with remimazolam (0.3 mg kg^−1^). The primary outcome was emergence time from anesthesia. Secondary outcomes included intravenous anesthesia induction time, total remimazolam dose, Visual Analog Scale (VAS) score at 30 min after awakening, and adverse events, including low pulse oximetry (SpO₂), bradycardia, hypotension, somatomotor response, and postoperative nausea and vomiting (PONV).

**Results:**

The AF-RMZ and F-RMZ groups had comparable demographic characteristics. AF-RMZ showed shorter induction (37.97 ± 4.38 s) and emergence times (68.40 ± 47.01 s) and reduced discharge time (36.38 ± 5.31 min) compared with F-RMZ, while total remimazolam use and postoperative 30-min VAS scores were similar. Heart rate and respiratory rate exhibited significant group-time interactions, whereas mean arterial pressure remained comparable. Peripheral oxygen saturation was transiently higher in the F-RMZ at early time points. All SpO₂ values remained clinically acceptable, supporting monitored respiratory safety. Adverse events were mild, with a lower incidence of somatomotor response in AF-RMZ (8.3% vs. 21.7%, *p* = 0.036).

**Conclusion:**

Alfentanil combined with remimazolam showed a slight advantage in anesthesia induction and emergence times, with comparable postoperative recovery, clinically stable hemodynamics, and safety profiles compared with fentanyl–remimazolam in outpatient gynecological procedures; however, the clinical impact of these differences may be limited.

**Clinical trial registration:**

https://www.chictr.org.cn, identifier ChiCTR2400094571.

## Introduction

Abortion is a widely recognized healthcare practice used for ending undesired pregnancies. Globally, first-trimester surgical abortion remains a common procedure, with significant implications for patient safety, comfort, and healthcare resource utilization. Ensuring optimal anesthesia during such procedures is crucial, as inadequate sedation or analgesia can increase perioperative stress, patient discomfort, and procedural inefficiency. For first-trimester surgical abortions, intravenous sedation is now the most commonly used method for vacuum aspiration, as it improves patient comfort and streamlines operating room efficiency (1). For any anesthetic technique to be effective, it needs to have a rapid onset, provide complete pain relief for the duration of the procedure, have limited impact on the circulatory and respiratory systems, and allow for quick recovery to ensure that the patient can be discharged as soon as possible. The selection and implementation of techniques greatly influence the performance of the anesthetic with regard to depth of sedation, analgesia, recovery, and overall safety in the perioperative period, which greatly affects the patient’s experience.

Remimazolam is an ultra-short-acting benzodiazepine with major indications for sedation during outpatient procedures due to its rapid onset of action. It is organ-independent with complete and rapid recovery. It also has mild effects and a good safety profile with respect to respiratory and cardiovascular function (2, 3). Alfentanil is a short-acting opioid analgesic that is suitable for short procedures and offers flexible and rapid dose titration. It achieves peak effect in 1–2 min and is associated with minimal adverse respiratory and hemodynamic effects (4). Alfentanil, when combined with remimazolam, theoretically offers considerable advantages. In particular, it may contribute to quicker anesthetic induction, shorter emergence times, and decreased somatomotor responses, possibly improving the efficiency of the entire perioperative process (5).

Previous studies have evaluated alfentanil or fentanyl individually or with other sedatives in short gynecological or outpatient procedures. However, there remains a lack of direct evidence comparing the AF-RMZ and F-RMZ combinations in first-trimester surgical abortion, particularly regarding clinically relevant outcomes such as induction time, emergence time, intraoperative somatomotor responses, and discharge readiness (5, 6).

Therefore, we hypothesize that the AF-RMZ combination will provide faster induction and emergence, reduce intraoperative somatomotor reactions, and maintain comparable safety profiles compared with F-RMZ in patients undergoing first-trimester surgical abortion. The objective of this research was to evaluate the clinical impact and safety of AF-RMZ and F-RMZ during first-trimester surgical abortion under intravenous sedation by assessing the primary outcomes of induction time, emergence time, and intraoperative somatomotor reactions, as well as the secondary outcomes of hemodynamic stability, remimazolam consumption, and postoperative pain during recovery.

## Materials and methods

### Patient inclusion and exclusion criteria

This study was a single-center, prospective, double-blind randomized controlled trial (RCT). The inclusion criteria were as follows: (1) aged 18–45 years; (2) gestational age ≤10 weeks; (3) American Society of Anesthesiologists (ASA) physical status I or II; (4) body mass index (BMI) 18.5–27.9 kg/m^2^; (5) scheduled for vacuum aspiration under intravenous anesthesia requiring intravenous anesthesia; and (6) no abnormalities in liver and kidney function prior to surgery.

The exclusion criteria were as follows: (1) patients with allergies or contraindications to opioid or benzodiazepine medications; (2) patients with a history of acute respiratory inflammation within 2 weeks without recovery; (3) patients with a history of poor recovery from previous surgical anesthesia; (4) electrolyte disturbances; and (5) concurrent use of other types of sedative or analgesic medications prior to surgery.

The withdrawal criteria were as follows: (1) patients with serious complications or accidents related to the operation or anesthesia during the perioperative period, and (2) patients who requested withdrawal during the study.

In the context of the need to design the study in a short period, no patients were involved in setting the research questions or the outcome measures, nor were they involved in developing plans for recruitment, design, or implementation of the study. No patients were asked to advise on the interpretation or writing of the results.

### Randomization and blinding

This study was a single-center, prospective, double-blind randomized controlled trial (RCT). A computer-generated random number table was used to allocate 60 participants to each group. Study drug preparation was performed by an independent anesthesiologist who did not participate in patient care, intraoperative management, or outcome assessment.

The intraoperative anesthesia was administered by a separate anesthesiologist who remained blinded to group allocation. Outcome data were collected by another anesthesiologist who was also blinded to the treatment assignment. Allocation concealment was maintained using sequentially numbered, sealed, opaque envelopes.

Patients, surgeons performing the procedure, and outcome assessors were all blinded to group allocation, thereby ensuring double-blinding of participants and outcome evaluators. Blinding integrity was maintained by preparing identical syringes for alfentanil and fentanyl labeled only with the participant identification number, preventing recognition of the administered opioid.

### Anesthesia and monitoring

All patients fasted for 8 h and refrained from drinking for 2 h prior to surgery. Peripheral veins were accessed after the patients arrived in the operating room, and electrocardiogram (ECG), heart rate (HR), respiratory rate (RR), pulse oximetry (SpO₂), and noninvasive blood pressure (NIBP) were monitored.

End-tidal CO₂ (EtCO₂) capnography was not routinely used due to procedural brevity (<10 min, typical for vacuum aspiration) and institutional protocols for low-risk cases; respiratory status was assessed via continuous SpO₂ (triggering intervention at ≤92%) and direct clinical observation by the attending anesthesiologist. While EtCO₂ monitoring is the gold standard, it was not used due to the very short duration of the procedure and low-risk patient population, and continuous SpO₂ monitoring with direct observation was deemed sufficient. Oxygen was administered via nasal cannula (5 L/min).

During the disinfection of the surgical site, the designated doses of either alfentanil (10 μg kg^−1^) or fentanyl (1 μg kg^−1^) were given according to group assignment. We selected these doses based on the established alfentanil/fentanyl equipotent ratio of approximately 10:1 reported in previous studies (7) and pilot data to achieve a similar analgesic effect while avoiding the more significant respiratory and hemodynamic side effects. This was followed by a bolus of remimazolam (0.3 mg kg^−1^) (8, 9). After the loss of consciousness and the disappearance of the eyelash reflex, the artificial abortion was performed without pain.

During the surgery, the cardiovascular parameters, such as heart rate and non-invasive blood pressure, were controlled and kept within normal ranges. If there were any changes beyond baseline non-invasive blood pressure and heart rate values of more than 30%, vasoactive medications were given. Respiratory depression was treated with jaw thrust, increased oxygen flow, and mask ventilation as indicated. Flumazenil and naloxone were kept on standby in the operating theater in case there was a need to reverse overdoses of sedatives or opioids causing respiratory depression. However, neither medication was needed for any patient.

An attending anesthesiologist assessed defined somatomotor responses, including purposeful movements of the upper or lower limbs, facial grimacing, or a combination of these, and a supplemental dose of remimazolam (2.5 mg) was administered at specific intraoperative intervals (T1–T4) without the use of other opioid medications for rescue analgesia.

For the purpose of ensuring inter-rater reliability, somatomotor responses were documented by two anesthesiologists for a subset of 20 patients, and Cohen’s kappa was calculated to be 0.85. In addition, the depth of sedation was also measured by the MOAA/S scale during the same time intervals (T0–T4) whenever this information was available.

Once the surgery was complete, the patient was monitored until they reached a stable recovery state, at which time they were transported to the post-anesthesia care unit (PACU) of the ambulatory surgical center, where monitoring continued until an Aldrete score ≥9 was achieved.

### Outcome measures

The primary outcome pertained to the emergence time from anesthesia, beginning from the end of the surgery until the patients spontaneously opened their eyes, while the secondary outcomes included the time of anesthesia induction and the total dose of remimazolam infused.

Time to anesthesia induction was defined as the time from remimazolam administration to complete loss of consciousness, while the total dose of remimazolam infused was defined as the total amount of remimazolam administered within the procedure time.

Further secondary outcomes post-surgery included the VAS pain score recorded at 30 min and the occurrence of low SpO₂ (intraoperative SpO₂ <95%) and the presence of bradycardia (intraoperative HR <60 bpm), hypotension (intraoperative MAP <65 mmHg), somatomotor response, and postoperative nausea and vomiting (PONV).

Measurements of systolic blood pressure (SBP), diastolic blood pressure (DBP), mean arterial pressure (MAP, MAP = SBP + 2 × DBP/3), heart rate (HR), respiratory rate (RR), and blood oxygen saturation (SpO₂) were collected at five specified time points: prior to induction of anesthesia (T0), at disappearance of eyelash reflex (T1), at cervical dilation (T2), during negative intrauterine pressure suction (T3), and after the surgery (T4).

### Statistical analysis

This study compared the effectiveness of alfentanil combined with remimazolam (AF-RMZ) and fentanyl combined with remimazolam (F-RMZ) in a double-blind randomized controlled trial. The primary endpoint was the emergence time from anesthesia.

Based on the observed variability of emergence duration from reviewed studies and internal databases, the averages for both groups were expected to differ by approximately 28 s with a standard deviation of 55 s. With a two-sided alpha of 0.05 and an expected power of 80%, the calculated sample size was 54 patients for each group. Given a 15% expected loss due to refusal or loss to follow-up, we aimed for 60 complete participants in each group to ensure sufficient power to detect emergence time differences.

Analysis was conducted using IBM SPSS Statistics for Windows Version 27. Full trial protocol and prespecified statistical analysis plan are available at ChiCTR2400094571. A pre-enrollment protocol modification was made, in which the remimazolam dose was increased from the registered 0.25 mg kg^−1^ to 0.3 mg kg^−1^, following pilot testing that demonstrated inadequate sedation, with multiple patients requiring supplemental dosing. This change was implemented prior to the enrollment of the first participant and had no impact on the primary outcomes, statistical analysis plan, or overall study validity.

All repeated measurements of HR, MAP, SpO₂, and RR were analyzed using a LMM with Bonferroni adjustment for multiple comparisons across time points. LMM was specifically chosen over repeated-measures ANOVA or MANOVA due to its flexibility with unbalanced repeated measures and lack of sphericity assumptions. No missing data occurred for primary outcome. The LMM accommodated all repeated measures without imputation, and all references to repeated-measures ANOVA have been removed for methodological consistency. Estimated marginal means ± standard error is reported, and 95% confidence intervals were calculated for all outcomes to provide a measure of clinical relevance. De-identified participant data, statistical code, and data dictionary available from corresponding author upon reasonable request.

## Results

**Figure 1:**
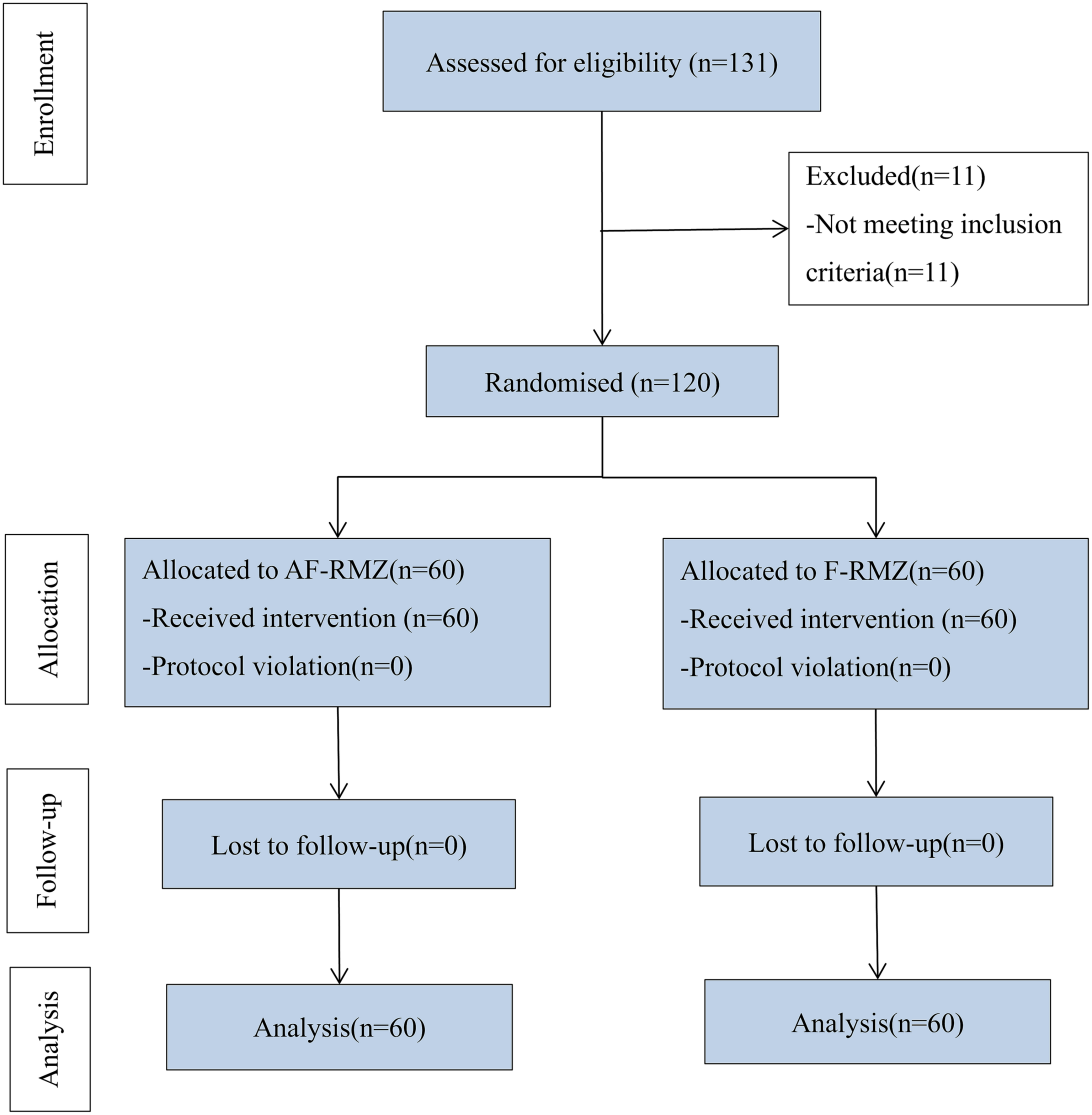
Flow diagram of the study.

A total of 131 patients were screened for eligibility between January 1, 2025 and June 30, 2025. Of these, 11 did not meet the inclusion criteria, while 120 were successfully recruited and randomized into either the AF-RMZ group or F-RMZ group (*n* = 60 per group). All 120 randomized patients were included in the final analysis, as shown in Figure 1.

Table 1 presents the demographic data of the patients. No statistically significant differences were observed between the groups.

**Table 1:** Demographic characteristics for each group.

Parameters	AF-RMZ (*n* = 60)	F-RMZ (*n* = 60)	Effect size	*p*-value
Age, year	32.65 ± 5.42	30.73 ± 5.93	1.92 (−0.14 to 3.97)	0.067
Height, cm	158.77 ± 4.99	160.50 ± 5.56	−1.73 (−3.64 to 0.18)	0.075
Weight, kg	54.94 ± 5.75	55.11 ± 6.33	−0.17 (−2.35 to 2.02)	0.880
BMI, kg/m^2^	21.82 ± 2.35	21.43 ± 2.34	0.39 (−0.46 to 1.24)	0.361
Duration of operation, min	5.24 ± 1.23	5.27 ± 1.08	−0.25 (−0.44 to 0.40)	0.907

The duration from surgery completion to regaining consciousness was notably shorter in the AF-RMZ group than in the F-RMZ group (68.40 ± 47.01 s vs. 96.07 ± 63.42 s, *p* < 0.001). Complete primary outcome data were available for all 120 randomized patients, with 60 participants analyzed per group. Additionally, the interval from remimazolam administration to loss of consciousness during anesthesia induction was significantly shorter in the AF-RMZ group than in the F-RMZ group (37.97 ± 4.38 s vs. 51.48 ± 5.42 s, *p* < 0.001).

There were no significant differences in total remimazolam consumption or postoperative 30-min VAS pain scores between the two groups. Notably, the AF-RMZ group exhibited significantly shorter discharge times than the F-RMZ group, with times of 36.38 ± 5.31 min vs. 40.65 ± 4.93 min (*p* < 0.001), indicating faster overall recovery after surgery, indicating faster overall recovery after surgery among the complete cohort of 60 patients per group, as displayed in Table 2.

**Table 2:** Primary and secondary anesthesia outcomes and postoperative recovery for AF-RMZ and F-RMZ groups.

Parameters	AF-RMZ (*n* = 60)	F-RMZ (*n* = 60)	Effect size	*p*-value
Primary outcome				
The emergence time from anesthesia, s	68.40 ± 47.01	96.07 ± 63.42	−27.67 (−47.87 to −7.47)	0.008
Secondary outcomes				
The induction time for intravenous anesthesia, s	37.97 ± 4.38	51.48 ± 5.42	−13.52 (−15.30 to −11.74)	<0.001
Total remimazolam, mg	16.80 ± 2.01	17.29 ± 2.70	−0.49 (−1.35 to 0.37)	0.260
Postoperative VAS score at 30 min	1.62 ± 0.69	1.82 ± 0.72	−0.20 (−0.46 to 0.06)	0.125
Discharge time (min)	36.38 ± 5.31	40.65 ± 4.93	−4.27 (−6.12 to −2.41)	<0.001

The interaction of group and time on heart rate and respiratory rate, as seen in Table 3 (*p* = 0.002 and *p* < 0.001, respectively), suggests that the time-series patterns of these metrics differ between the alfentanil and fentanyl groups. These differences across time were analyzed with a multilevel linear mixed model with Bonferroni adjustments to control for Type I errors. Both groups showed an increase in HR over time, but the fentanyl group had higher values at the later measurement time points. There was a decrease in the respiratory rate (RR) after anesthesia induction, and the rate then gradually increased after the reduction at T1, as seen in the alfentanil group. In the fentanyl group, the mean arterial pressure (MAP) appeared to be higher (particularly at the T2 time point) than in the alfentanil group. However, the group–time interaction was not statistically significant (*p* = 0.077), indicating that both groups likely followed similar time trends. In peripheral oxygen saturation (SpO₂), a statistically significant group–time interaction was present (*p* < 0.001), and it was also noted that the fentanyl group had slightly higher oxygen saturation (SpO₂) than the alfentanil group during time intervals T1–T2, and that adequate oxygenation was present in all study participants during the study intervals.

**Table 3:** Hemodynamic and respiratory parameters over time in AF-RMZ and F-RMZ.

Variable	Group	T0	T1	T2	T3	T4	*p*-Value
HR	AF-RMZ	76.42 ± 1.14	82.70 ± 1.20	82.47 ± 1.24	79.83 ± 1.23	84.12 ± 1.51	0.002
	F-RMZ	78.28 ± 1.14	81.33 ± 1.20	81.10 ± 1.24	81.25 ± 1.23	86.45 ± 1.51	
MAP	AF-RMZ	84.17 ± 1.23	71.23 ± 1.00	74.72 ± 1.21	78.03 ± 1.26	78.38 ± 1.26	0.077
	F-RMZ	87.32 ± 1.23	76.43 ± 1.00	76.45 ± 1.21	81.10 ± 1.26	81.68 ± 1.26	
SpO₂	AF-RMZ	98.55 ± 0.09	97.80 ± 0.23	97.97 ± 0.20	98.63 ± 0.12	98.78 ± 0.10	<0.001
	F-RMZ	98.58 ± 0.09	98.68 ± 0.23	98.13 ± 0.20	98.35 ± 0.12	98.62 ± 0.10	
RR	AF-RMZ	16.23 ± 0.21	9.87 ± 0.17	11.30 ± 0.14	12.48 ± 0.12	14.20 ± 0.14	<0.001
	F-RMZ	16.17 ± 0.21	11.72 ± 0.17	11.30 ± 0.14	12.72 ± 0.12	14.08 ± 0.14	

HR, heart rate; MAP, mean arterial pressure; SpO₂, peripheral oxygen saturation; RR, respiratory rate. Values are estimated marginal means ± standard error.

**Figure 2:**
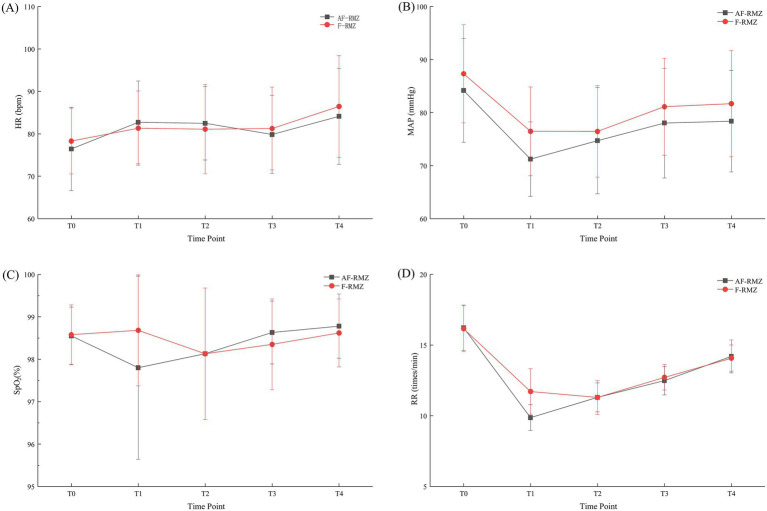
Temporal changes in HR **(A)**, MAP **(B)**, SpO_2_ **(C)**, and RR **(D)** in the AF-RMZ and F-RMZ groups. Data are presented as mean ± SE, with shaded areas representing 95% confidence intervals (CIs). T0, before anesthesia induction; T1, upon disappearance of the eyelash reflex; T2, at cervical dilatation; T3, during intrauterine negative pressure suction; T4, upon completion of the operation. Statistical analysis was performed using repeated-measures ANOVA to assess differences over time between groups.

Multiple variables, including heart rate, mean arterial pressure, respiratory rate, and peripheral oxygen saturation, were assessed continuously and simultaneously across five predetermined time intervals: T0, T1, T2, T3, and T4. There were no notable differences (*p* > 0.05) in heart rate, mean arterial pressure, and peripheral oxygen saturation between the two groups (AF-RMZ and F-RMZ) across T0, T2, T3, and T4. At T1, which was marked by the absence of the eyelash reflex, the mean arterial pressure, peripheral oxygen saturation, and respiratory rate were significantly different (*p* < 0.05), with AF-RMZ values lower than F-RMZ. The groups were not statistically different in heart rate. Average vital signs during the course of the procedure were also statistically and clinically acceptable across both groups. The standard error (SE), mean values, and 95% confidence intervals are shown in Table 3 and Figure 2, where the confidence intervals or means are represented by shaded regions. Repeated measurements across time were analyzed using a linear mixed-effects model with Bonferroni adjustment, consistent with the statistical methods described above.

The adverse events exhibited by patients receiving alfentanil and fentanyl are shown in Table 4. Patients in the alfentanil group and fentanyl group exhibited body movement in 8.3 and 21.7%, respectively. Meanwhile, hypoxemia was present in 11.7 and 5.0% of patients, hypotensive episodes were documented in 11.7 and 6.7%, and postoperative nausea and vomiting (PONV) was present in 6.7 and 8.3% of patients in the alfentanil and fentanyl groups, respectively.

**Table 4:** Incidence of adverse events and associated effect estimates in alfentanil-remimazolam (AF-RMZ) versus fentanyl-remimazolam (F-RMZ) groups.

Adverse event	AF-RMZ (*n* = 60)	F-RMZ (*n* = 60)	Risk ratio (95% CI)	Odds ratio (95% CI)	Fisher’s exact *p*
Somatomotor response	5 (8.3%)	13 (21.7%)	0.38 (0.15–0.97)	0.33 (0.11–0.99)	0.036
Low SpO_2_	7 (11.7%)	3 (5.0%)	2.33 (0.63–8.59)	2.51 (0.62–10.21)	0.322
Hypotension	7 (11.7%)	4 (6.7%)	1.75 (0.52–5.88)	1.85 (0.51–6.68)	0.529
PONV	4 (6.7%)	5 (8.3%)	0.80 (0.22–2.91)	0.79 (0.20–3.08)	1.000
Bradycardia	0 (0)	0 (0)			

Data represent numbers (percentages) of patients experiencing each adverse event among 60 participants per group. All 120 randomized patients contributed to adverse event analyses. Risk ratios and odds ratios with 95% confidence intervals were calculated using Mantel–Haenszel methods. Bradycardia defined as HR <60 bpm intraoperatively.

## Discussion

Surgical abortions are common clinical procedures where appropriate analgesia ensures patient comfort and procedural efficiency (10). This research directly contrasted AF-RMZ versus F-RMZ combinations in first-trimester surgical abortion. Key findings demonstrated advantages of AF-RMZ in shortening induction and emergence times, improving somatomotor response control, and maintaining hemodynamic stability during the procedure.

Our study contributes unique evidence regarding the use of alfentanil with remimazolam during first-trimester surgical abortion, with outpatient recovery endpoints (emergence and discharge) included in the analysis. Prior studies have demonstrated alfentanil-remimazolam efficacy during outpatient and daytime surgeries. We have added surgical abortion to this growing list of procedures (5, 11).

These time advantages align with the pharmacokinetic profile of alfentanil. Alfentanil is a short-acting *μ*-opioid receptor agonist with rapid onset and peak effect, a small volume of distribution, and a short elimination half-life, resulting in a brief duration of action compared with fentanyl and sufentanil (12). Remimazolam is an ultra-short-acting benzodiazepine characterized by rapid onset, organ-independent metabolism, and rapid recovery, with minimal circulatory and respiratory depression (13, 14). Thus, remimazolam is a promising option for anesthesia in outpatient and daytime surgery (15).

Although the AF-RMZ group had a faster onset of action and earlier recovery, VAS scores and remimazolam consumption were comparable, confirming equivalent analgesia despite AF-RMZ speed advantages. Although the absolute difference in emergence time (30 s) between groups was modest, similar findings in other outpatient anesthesia studies have shown that optimized sedative-opioid combinations (e.g., alfentanil with remimazolam) can lead to meaningful improvements in induction and recovery profiles, which may enhance overall perioperative efficiency in high-throughput ambulatory settings (16, 17). This supports the conclusion that alfentanil can successfully suppress surgical stimulation and satisfy the pain relief requirements for surgical abortion (18). It is relevant to this study that discharge time is clinically relevant, which adds weight to the utility of the study’s findings.

Alfentanil combined with remimazolam has been shown in clinical studies to have fewer deleterious effects on the cardiovascular system and to provide better hemodynamic stability (8, 19, 20). There were comparable and stable trends in heart rate, MAP, and SpO₂ in the perioperative period in the two groups of our investigation. The changes in each group were the result of the interaction of the surgical procedure and the anesthesia, with no relevant variations between the two groups at many measured times.

In relation to the aforementioned case, at the time when the eyelash reflex disappeared (T1), which represents when the peak effect of the drug would be expected, the MAP and SpO₂ values of the AF-RMZ group were lower compared to the values of the F-RMZ group. This is explained by the fact that alfentanil reaches its anesthetic peak effect earlier, and hence is more likely to exert its anesthetic effect at that time (21). It is important to clarify that this difference was only temporary and specific to T1, and was safety-neutral, as it did not include any significant increase in adverse events, i.e., hypotensive events and hypoxemia.

Lack of sufficient analgesia or sedation during surgeries often results in somatomotor responses, which influence both the surgical procedure and may add complications (22). One of the most notable findings of the current study was the reduced occurrence of intraoperative movement in the AF-RMZ group versus that in the F-RMZ group, which demonstrates better intraoperative analgesia and sedation. This improvement was most likely the sum of the effects of both alfentanil which has effective analgesia and remimazolam which sedates by interacting with gamma-aminobutyric acid (GABA) receptors, specifically GABA_A (23). Alfentanil has analgesic effects through the activation of the *μ*-opioid receptor (MOR). Since GABA_A receptors (GABAAR) and MOR are co-expressed in some primary afferent neurons, the use of alfentanil and remimazolam together is likely to act on the same circuitry, amplifying sedation effects (24). This integrated method blunts patient responses to surgical triggers like cervical dilation and uterine suctioning, directly improving the procedure’s safety and effectiveness.

This study confirms that the combination of remimazolam and alfentanil is a useful anesthetic protocol for surgical abortion. The protocol has real utility in that the induction time, emergence time, and discharge time all reached statistical significance. The protocol is extremely useful from a perioperative perspective. The protocol permits rapid induction and recovery, stable hemodynamics, adequate analgesia, decreased intraoperative movement, and is associated with a lower incidence of adverse effects such as respiratory depression, hypotension, bradycardia, and PONV (25, 26). These attributes make the regimen particularly suitable for outpatient and day-surgery anesthesia, where rapid recovery, efficiency, and safety are essential (27–29).

Notable limitations exist in this particular study. To begin with, the study was completed at a single center. As such, findings may not be generalizable. Validation will require multicenter studies. Additionally, the modest sample size may have limited the detection of rare adverse events and may have decreased the power for subgroup analyses. Pain following surgery was only evaluated once 30 min after the procedure, using the VAS, and this may not reflect later pain, such as that caused by uterine cramping. Likewise, it was not possible to use objective depth-of-anesthesia monitoring, which may have impacted the assessment of somatomotor responses. Lastly, Aldrete scoring was used to guide discharge. However, perioperative efficiency conclusions could be strengthened with a more thorough assessment of discharge readiness.

End-tidal CO₂ capnography was not routinely employed in this study, with respiratory status assessed via SpO₂ and direct clinical observation by the attending anesthesiologist. While no clinically significant respiratory depression occurred, this approach may underestimate hypoventilation risk, particularly with opioid–benzodiazepine combinations. EtCO₂ monitoring provides earlier detection of hypercapnia than SpO₂ alone; its absence is a notable limitation, especially in higher-risk outpatient sedation. Future studies should incorporate nasal capnography for comprehensive respiratory profiling.

## Conclusion

First-trimester surgical abortion can be performed safely and effectively using both alfentanil-remimazolam and fentanyl-remimazolam regimens. The alfentanil-remimazolam combination demonstrated a slight advantage in induction and emergence from anesthesia, with modestly reduced recovery times and a lower incidence of intraoperative somatomotor responses. Hemodynamic and respiratory parameters remained stable under continuous SpO₂/clinical monitoring, in both groups, and the overall safety profiles were comparable. Although statistically significant, the observed differences in induction and emergence times may have limited clinical relevance in routine practice. Overall, alfentanil-remimazolam represents a viable and efficient anesthetic option for outpatient surgical procedures without conferring a clear superiority in clinical outcomes.

## Data Availability

The original contributions presented in the study are included in the article/supplementary material, further inquiries can be directed to the corresponding author.
